# Examining disgust learning through category conditioning: Evidence from trial-unique presentations and oculomotor avoidance

**DOI:** 10.3758/s13420-025-00669-0

**Published:** 2025-03-10

**Authors:** Sinem Söylemez, Aycan Kapucu

**Affiliations:** 1 Manisa Celal Bayar University, Manisa, Turkey; 2 Ege University, İzmir, Turkey

**Keywords:** Disgust, Category conditioning, Oculomotor avoidance, Evaluative conditioning

## Abstract

Disgust is a basic emotion that motivates avoidance behaviors to protect organisms from pathogens. Objects of disgust are acquired through classical conditioning mechanisms. Oculomotor avoidance serves as an objective marker of disgust, yet previous studies have relied on repeated presentations to establish disgust conditioning. This study aimed to adapt the category-conditioning paradigm (Dunsmoor et al., *Cerebral Cortex*, *24*, 2859-2872, 2014) for disgust learning by employing trial-unique presentations, offering a novel tool for future research. In our experiment, items of two categories – furniture and vehicles – were paired with either disgusting or neutral scenes. Participants' eye movements were tracked, and self-reported measures were collected. The results demonstrated that the category-conditioning task with trial-unique stimuli effectively induced oculomotor avoidance. Participants exhibited both unconditioned avoidance responses to disgusting scenes and conditioned avoidance responses to category items associated with disgust. Eye-tracking data further revealed that disgust-associated stimuli motivated avoidance beyond their role as mere predictors of an aversive stimulus. Interestingly, participants initially exhibited a tendency to view the disgusting image before engaging in avoidance behavior. In conclusion, this study demonstrates that the adapted category-conditioning paradigm successfully elicits conditioned responses using trial-unique stimuli. We believe that this paradigm will provide a valuable tool for future research on disgust learning.

## Introduction

Disgust functions as a fundamental emotional response within the human repertoire, serving pathogen avoidance (Rozin & Fallon, 1987). This emotion is intricately linked to the behavioral immune system (BIS) – a constellation of psychological mechanisms postulated to safeguard organisms from environmental pathogens and infectious agents without direct physical contact (Curtis, 2011; Murray & Schaller, 2016; Schaller & Park, 2011). The operational framework of disgust hinges on the concept of "magical thinking" (Rozin & Nemeroff, 1990), a theoretical construct encompassing two key sub-systems: *contagion* and *similarity*. Within this framework, any stimuli contacted with, or bearing a resemblance to, a known disgusting stimulus can elicit disgust. Empirical evidence supporting this notion is exemplified by research demonstrating avoidance behavior towards cookies visually resembling canine feces, or sterilized cockroaches introduced into a potable water source (Rozin & Nemeroff, 1990).

Although disgust is a basic emotion (Ekman, 1992), the variety of its elicitors across cultures and contexts can be understood by exploring its connection to learning mechanisms. The objects eliciting disgust are learned via classical conditioning mechanisms (Rozin & Fallon, 1987; Söylemez & Kapucu, 2024a). However, disgust learnings have been suggested to differ from other classical conditionings (i.e., signal learning or expectancy learning) as it depends on *evaluative conditioning* (Armstrong et al., 2014, 2019; Armstrong & Olatunji, 2012). Evaluative conditioning is an associative type of learning that is not just about learning the relationship between the two stimuli (De Houwer et al., 2001). In evaluative learning, the conditioned stimulus (CS) and the unconditioned stimulus (US) are intertwined and integrated (Olatunji et al., 2007; Palombo et al., 2021) and form a unified stimulus. As a result, the hedonic value of the US shifts to the CS, and the CS gains the same emotional value as the US (Apicella et al., 2018). In expectancy learning, a CS activates both the memory of the US and an expectation of its occurrence, whereas in evaluative conditioning, the CS activates only the memory of the US, playing a key role in the acquisition of emotional responses (Köksal et al., 2017). Therefore, neutral stimuli associated with disgust are expected to acquire disgust value themselves and elicit oculomotor avoidance. For example, a phone that falls into the toilet and is thoroughly cleaned afterward still elicits disgust – not due to the expectation of fecal contamination, but because it is now perceived as inherently disgusting.

In classical conditioning studies, an unconditioned response (UR) to a US is observed, and it is demonstrated that participants exhibit a new response (CR; conditioned response) to a stimulus that becomes associated with the US after repeated pairings (Pavlov, 1927; Pearce & Bouton, 2001; Rescorla, 1988). Thus, disgust conditioning requires an objective response to be studied. Disgust is associated with the “fast avoidance” behavioral pattern (Curtis et al., 2011) and this avoidance behavior can manifest itself in many ways (see Shook et al., 2019), one of which is *attentional avoidance*. Given the concrete perceptual properties of disgust-related stimuli (Royzman & Sabini, 2001), it seemed appropriate to investigate attentional avoidance with eye tracking. Previous eye-movement measures were repeatedly shown to provide good internal consistency and test-retest reliability for disgust avoidance (Armstrong et al., 2021). Classical conditioning studies on disgust using eye tracking have shown that *oculomotor avoidance* (i.e., avoidance of looking at a particular area) can be an objective measure of an unconditioned disgust response (Armstrong et al., 2014, 2019, 2022a, b; Dalmaijer et al., 2021; Mason & Richardson, 2010). In addition, oculomotor avoidance could occur as a CR to disgust-associated neutral stimuli (Armstrong et al., 2014, 2019).

In typical conditioning experiments, CS and US are repeatedly paired to enhance the likelihood of establishing a conditioned response. The repeated presentation of stimuli in conditioning studies may hinder the assessment of other cognitive processes within the same experimental framework. That is, the repeated presentation of stimuli limits the measurement to a single CR, making it difficult to assess other cognitive mechanisms, such as memory. Memory studies typically involve trial-unique presentation of stimuli, followed by a memory test after a certain time interval, in order to assess how well these stimuli are remembered (e.g., Mather & Knight, 2008; Söylemez et al., 2021; Yüvrük et al., 2023). However, studying different cognitive processes (e.g., learning and memory) together could enrich emotional learning studies (Dunsmoor & Kroes, 2019). To achieve this, we adapted the category-conditioning procedure (Söylemez & Kapucu, 2024b), originally developed by Dunsmoor and colleagues (Dunsmoor & Kroes, 2019; Dunsmoor et al., 2014, 2015; Patil et al., 2017). In this study, we aimed to test the disgust conditioning task using trial-unique presentations, enabling future research to explore disgust learning from various perspectives, such as memory and attention. The primary goal of the present study was to evaluate the effectiveness of this paradigm in facilitating trial-unique presentations within disgust conditioning.

### Current study

In the original version of category conditioning, pictures of animals and tools are used. Different members of the same category are presented once as CS^+^ (paired with US^+^) or CS^-^ (not paired with US^+^), where the US is mild electrical shock. Since each categorical object picture is presented only once, every picture represents a distinct and isolated event during conditioning. The neurobehavioral mechanisms underlying category conditioning were shown to be the same as those underlying classical conditioning based on simple repetitive CS presentation (Dunsmoor & Kroes, 2019). We aimed to test this paradigm for disgust; when we consider contagion and similarity characteristics of disgust, conditioning is expected to be successful at the category level. In the adapted version of this paradigm for the present study, furniture and vehicle categories were used as CS, and disgust images were used as US^+^. Eye tracking was used to test whether oculomotor avoidance appears as UR and CR. We hypothesized that disgust-associated stimuli would gain disgust value (Söylemez & Kapucu, 2024b) and lead to oculomotor avoidance as a CR through the category-conditioning procedure. We aimed to improve a disgust-conditioning paradigm by using trial-unique presentation. For that purpose, a previous eye-tracking study (Armstrong et al., 2019) that showed *oculomotor avoidance* as both a CR and a UR to disgust was taken as reference and adapted to category conditioning.

This study was approved by the Institutional Review Board of Ege University (Approval Number: 929). All data, supplementary materials, and ethics approval are publicly available via the Open Science Framework at: https://osf.io/rp8gc/

## Method

### Participants

Forty-five university students participated in the study. Six participants were excluded due to problems that occurred during the eye-tracking procedure (e.g., quitting the study, physical discomfort), resulting in a final 39 participants (72% female; *M*_*age*_* =* 19.54 years, *SD* = 1.35 years). G*Power analysis (Faul et al., 2007), conducted using the average fixation duration values for CS^+^ and CS^-^ reported in Armstrong and colleagues (2019), showed that a sample size of 26 is adequate for detecting a significant effect size (*d*_*z*_ = .74), with α = .05 and power = .95 for a paired-sample t-test. Given the use of distressing images, the requirement for participants to remain stationary for extended periods, and the introduction of trial-unique presentations not employed in previous studies (e.g., Armstrong et. al., 2019, 2022a, b; Dalmaijer et al., 2021), the sample size was deliberately set slightly above the minimum required to ensure robustness and accommodate potential dropouts. All participants received course credits in exchange for their participation.

### Materials

#### Stimuli

All US and CS stimuli were taken from Söylemez and Kapucu’s (2024b) study. Thirty disgusting (*M*_disgust_ = 6.27, *SD* = 0.22) and 30 neutral (*M*_neutral_ = 1.36, *SD* = 0.28) scene images^1^ were used as US^+^ and US^-^. Disgust images consist of pictures containing body waste, maggots, and decayed food.^2^ Disgust stimuli set did not include the images of blood or injury because basic disgust and blood-injury disgust are associated with different psychophysical processes (Chapman & Anderson, 2012). Basic disgust is associated with nausea and abdominal contractions (Harrison et al., 2010; Stern et al., 2001), while blood-injury disgust is linked to dizziness and fainting (Cisler et al., 2009a).

Sixty object images from each of the vehicle and furniture categories were used as CS. (see Dunsmoor et al., 2014; Söylemez & Kapucu, 2024b). These studies showed that the selected images significantly differed in typicality^3^ for vehicle (*M =* 7.36, *SD* = 1.79) and furniture (*M =* 8.33, *SD* = 1.79) categories (*t*(118) = −2.94, *p* < .01, Cohen’s* d* = .541). We balanced category-emotion pairs across participants to control the typicality effect. We chose different categories to the ones used in the original paradigm (tools and animals; Dunsmoor et al., 2014) for various reasons. First, Dunsmoor and colleagues (2014) suggested that the animal category is associated with more specific mental processes than the tool category, even though they are given the same behavioral responses after conditioning, probably for evolutionary reasons. In addition, some studies have suggested that disgust is associated with animalness (Angyal, 1941; Rozin & Fallon, 1987). Thus, using animals as the neutral CS category would be confounding. Second, the tools used in the original paradigm were not all commonly used objects in Turkey, which posed a problem since the basic level of categories is the first-learned level during language development and sensitivity to culture (Hajibayova, 2013). Thus, we chose furniture and vehicle categories since both are equally relevant to daily life and would be familiar to participants (university students). Finally, we developed a larger set of stimuli for the CS to enhance the diversity of categorical objects and enable trial-unique presentations (for further information about US and CS stimuli, see Söylemez & Kapucu, 2024b, https://osf.io/wbgvr/).

#### Category conditioning

This paradigm was used to associate neutral categorical objects with disgusting or neutral scene images. For this purpose, different objects from one category were matched with various disgusting scene images (thus becoming CS^+^), while the objects from the other category were matched with neutral scene images (thus becoming CS^-^). Each CS image is transformed into the matched US image to increase the CS-US contingency (Armstrong et al., 2019) through FantaMorph Deluxe 5 (Abrosoft, NE, USA). This way, CS and US overlapped during the morphing period; this temporal arrangement was shown to provide the strongest and fastest conditioning (Mazur, 2017, *p*. 69). Examples of CS-US pair morph videos can be found at https://osf.io/rp8gc/. Sixty morph videos, each lasting 14 s, were created in which a CS appeared for the first 6 s, transformation occurred for 2 s, and a US appeared for the last 6 s. While the objects from one category were followed by disgusting scenes (disgust pair), the objects from the other category were followed by neutral scenes (neutral pair). The category-emotion type matching was balanced across participants. Moreover, four different lists were created to vary CS-US pairs used for morph videos.

**Eye movements:** Eye movements were recorded at a frequency of 1,000 Hz employing EyeLink 1000+ eye tracker, manufactured by S.R. Research LTDC in Canada, with a spatial accuracy of less than 1/4th of a degree. The commencement and conclusion of saccades were identified automatically using a default velocity criterion of 30 degrees per second, as implemented in the EyeLink software. Stimuli were displayed on a 24-in. monitor at a distance of 90 cm with a refresh rate of 144 Hz, and the experiment was run using Experiment Builder software. A chin rest and forehead support were employed to ensure head-position stability. Binocular viewing was employed, but the data analysis focused exclusively on the dominant eye. Individual participants were calibrated using a 9-point matrix covering the screen dimensions of 1,920 × 1,080 pixels. The stimulus pairs were displayed side by side in a 16.6 × 12.9 cm size on the right and left side of the screen. Participants gazed at each calibration point in sequence and then performed a validation procedure to confirm that fixation was within 0.5° of the calibration points.

Interest Area Dwell Time refers to the average duration of all fixations made on the areas of interest. This viewing time was calculated separately for each trial of each stage *pre-conditioning*, *US exposure*, *conditioning*, and *post-conditioning* for each stimulus (CS^+^, CS^-^, US^+^, and US^-^) and means were calculated. Since drift correction was applied between each trial during the experiment, the eye-tracking data were reliably collected. Therefore, it was found that, unlike Armstrong and colleagues' (2019) study, there was no need to exclude a trial from the total score if more than half of the trial was spent looking outside the areas of interest. During the conditioning phase, video stimuli were used, 1-s segment analysis was performed in these sections. Videos of 14 s were divided into 1,000-ms time bins, and fixations on CS^+^ and CS^-^ as well as US^+^ and US^-^ were compared for each segment. The data from the 2-s transition from CS to US during the conditioning phase was excluded from the analysis.

### Procedure

**Fig. 1:**
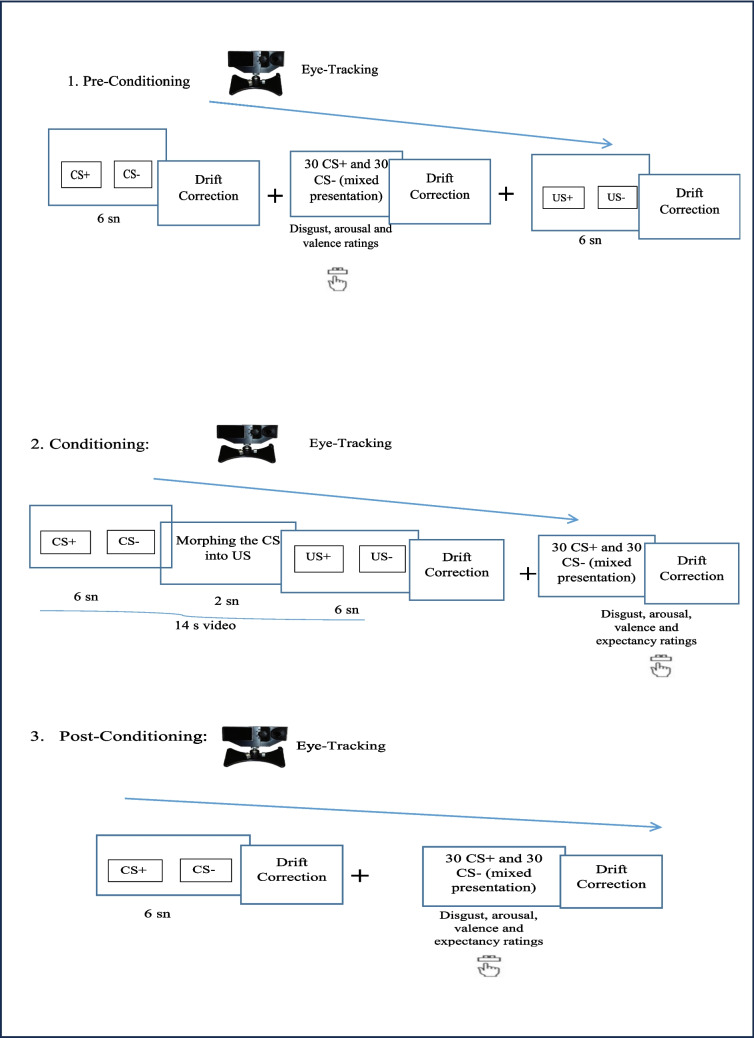
Stages of the experimental procedure. *CS* conditioned stimuli, *US* unconditioned stimuli

Participants were randomly assigned to one of four lists constructed with different CS-US image pairings. The participants were informed that the experiment measured pupil size to prevent them from consciously directing their eye movements (see Armstrong et al., 2019). The calibration and validation corrections were applied using the 9-point procedure on the eye-tracking device. Drift correction was applied after each trial, and participants were required to look at the fixation point in the middle of the screen and press the space bar to proceed to the next trial. This was done to ensure the reliable collection of eye-movement data and to prevent participants from passively looking at the screen. The participants underwent category-conditioning stages of *pre-conditioning*, *US exposure*, *conditioning*, and *post-conditioning (extinction)* (Fig. 1). Each stage included 30 unique trials in which CS^+^ and CS^-^ images were presented side by side for 6 s to allow for perceptual competition between stimuli. Thus, the CS images (CS^+^, CS^-^) or US images (US^+^, US^-^) were presented simultaneously on the right and left sides of the screen. The position and order of the stimuli were randomized.

During the *pre-conditioning stage*, we recorded baseline fixation durations and conducted pre-test evaluations for the CS images. Participants rated each CS on disgust, arousal, and valence; using a 9-point scale (arousal: 1 = "very low" to 9 = "very high"; valence: 1 = "very negative" to 9 = "very positive"; disgust: 1 = "very low" to 9 = "very high"). The *US exposure stage* included disgusting and neutral scenes that will be used as the US in the conditioning stage. Armstrong and colleagues (2019) suggested that US exposure before conditioning allows us to see CR faster without US devaluation (Experiment 2). No ratings were taken for the US at the end of this stage. Next, in the *conditioning stage*, 14-s morph videos (disgust pair and neutral pair) were presented side by side on the screen. One hundred percent contingency was used (see Armstrong et al., 2019; Mason & Richardson, 2010). Between trials participants were required to fixate on the fixation point in the middle of the screen and press the space bar to move on to the next trial. When the conditioning stage is completed, participants rated each CS for disgust, arousal, valence, and US expectancy. US expectation evaluations were used to test participants’ awareness of the CS-US contingency.^4^ Finally, the *post-conditioning (extinction) stage* was the same as the pre-conditioning stage, except that US expectancy ratings were also taken at the end of this stage. The whole experiment lasted about 60 min.

### Data analysis

Mean self-report ratings and Interest Area, Dwell Time scores, were tested as dependent variables. Dwell time (i.e., overall fixation duration) was calculated and averaged for each stimulus (CS^+^, CS^-^, US^+^, US^-^), separately for each stage, to be used as an indicator of oculomotor avoidance. A shorter dwell time indicates greater oculomotor avoidance. Across different analyses, CS^+^ was compared with CS^-^, and US^+^ was compared with US^-^ in terms of dwell times. Consistent with previous studies (Armstrong et al., 2019; Lissek et al., 2008; Mason & Richardson, 2010), paired-sample t-tests were used to compare these stimuli within each stage, and Bonferroni correction was applied to control for type I error in multiple comparisons.

**Fig. 2:**
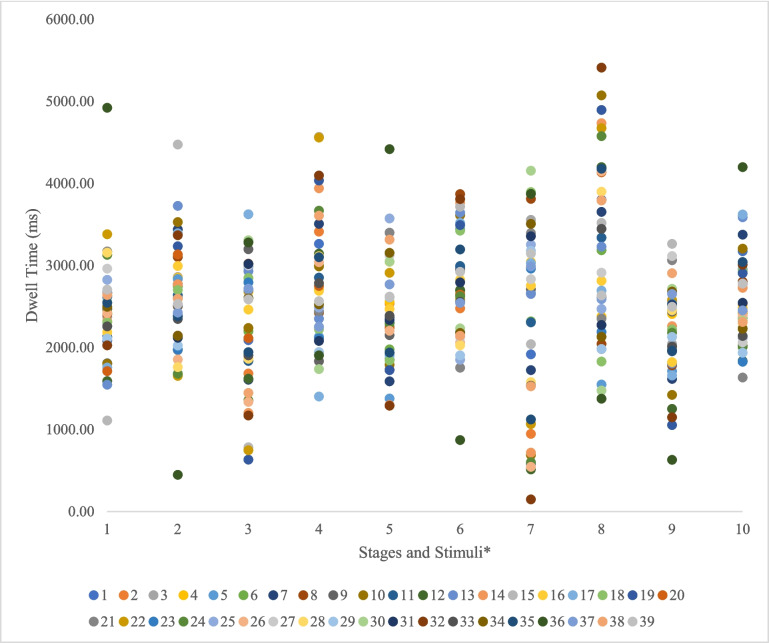
Individual oculomotor avoidance data as conditioned (CR) and unconditioned (UR) responses* 1. PreCond_CS^*+*^*, 2. PreCond_CS*^*-*^*, 3.USExposure_US*^*+*^*, 4. USExposure_US*^*-*^*, 5. Cond_CS*^*+*^*, 6. Cond_CS*^*-*^*, 7. Cond_US*^*+*^*, 8. Cond_US*^*-*^*, 9. PostCond_CS*^*+*^*, 10. PostCond_CS*^*-*^*.* See https://osf.io/9vc2s for more information and the disgust proneness score of each participant

Eye-movement data were analyzed to test whether oculomotor avoidance (measured by dwell time) was displayed as UR and CR to disgust (see Table 1 and 2 and Fig. 2). To test oculomotor avoidance as the UR, the US^+^ were compared to US^-^ at pre-exposure and conditioning stages. To test it as the CR, the CS^+^ were compared to CS^-^ at pre-conditioning, conditioning, and extinction stages. All data and supplementary materials have been made publicly available at https://osf.io/rp8gc/.

**Table 1:** Descriptive statistics for self-report ratings

	Pre-conditioning		Conditioning		Post-conditioning	
	CS^+^	CS^-^	CS^+^	CS^-^	CS^+^	CS^-^
Disgust	1.86 (0.81)	1.89 (0.97)	2.13 (1.50)	2.08 (1.36)	2.28 (1.84)	2.04 (1.13)
Valence	4.88 (0.60)	5.02 (0.65)	4.64 (0.81)	4.88 (0.84)	4.61 (0.86)	4.88 (0.80)
Arousal	3.49 (1.44)	3.58 (1.63)	3.27 (1.77)	3.51 (1.89)	3.22 (1.83)	3.37 (1.82)
Expectancy	-	-	3.62 (2.17)	2.90 (1.43)	3.62 (2.29)	2.83 (1.58)

*CS* conditioned stimulus

**Table 2:** Mean dwell times for each stage

	Dwell time (ms)			
	CS^+^	CS^-^	US^+^	US^-^
Pre-conditioning	2445.37 (630.41)	2591.53 (699.46)	-	-
US Exposure	-	-	2261.46 (793.96)	2807.44 (778.94)
Conditioning	2342.14 (708.58)	2731.51 (695.30)	2272.73 (1150.48)	3107.60 (1143.51)
Post-conditioning/Extinction	2139.83 (577.92)	2579.55 (554.61)	-	-

*Note*. Values represent the mean, while the values in parentheses represent the standard deviation

## Results

### Self-report measurements for conditioned stimulus (CS)

One participant’s data could not be recorded because they used the keyboard instead of the mouse to rate the stimuli. Self-report disgust ratings for CS^+^ and CS^-^ at each stage were compared with paired-sample t-tests (see Table 1). Bonferroni correction was applied (*p* = 0.0167). Results showed that ratings did not differ between these stimuli at pre-conditioning (*t*(37) = -.214, *p* = .832, Cohen's* d* =−0.035, 95% CI [−0.353, 0.284]), conditioning (*t*(37) = .27, *p* = .791, Cohen's* d* = 0.043, 95% CI [−0.275, 0.361]) or extinction stages (*t*(37) = .99, *p* = .330, Cohen's* d* = 0.160, 95% CI [−0.161, 0.479]) (Table 1). We also tested whether any differentiation occurred between CS^+^ and CS^-^ from pre-conditioning to extinction using a 3 (stage: pre-conditioning, conditioning, extinction) × 2 (CS type: CS^+^, CS^-^) repeated-measures ANOVA with Greenhouse-Geisser correction. This analysis aimed to examine how the differentiation between CS^+^ and CS^-^ evolved across stages (Armstrong et al., 2019). Results showed that neither stage (*F*(1.20, 44.23) = 1.955, *p* = .149, *η*_*p*_^2^ = .050) nor CS type (*F*(1, 37) = .264, *p* = .611, *η*_*p*_^2^ = .007) had a significant effect on the disgust ratings. The interaction effect was not significant, either (*F*(1.42, 52.54) = 1.251, *p* = .292, *η*_*p*_^2^ = .033).

For the valence ratings, CS^+^ and CS^-^ did not differ at pre-conditioning (*t*(37) = −1.273, *p* = .211, Cohen's* d* =−0.206, 95% CI [−0.527, 0.116]), conditioning (*t*(37) = −1.872, *p* = .069, Cohen's* d* = −0.304, 95% CI [−0.627, 0.024]), or extinction (*t*(37) = 1.902, *p* = .065, Cohen's* d* = −0.308, 95% CI [−0.632, 0.019]). Repeated-measures ANOVA test showed that stage had a significant main effect on the valence ratings (*F*(1.60, 59.32) = 3.544, *p* =.045, *η*_*p*_^2^ = .087), as all CS images were rated as increasingly more negative from pre-conditioning to extinction. In addition, CS type had a significant main effect (*F*(1, 37) = 4.328, *p* = .044, *η*_*p*_^2^ = .105), with CS^+^ rated more negatively than CS^-^. Again, the stage and CS type interaction was not significant (*F* <1).

For the arousal ratings, paired-sample t-test results showed that CS^+^ and CS^-^ did not differ at pre-conditioning (*t*(37) = -.309, *p* = .759, Cohen's* d* = −0.050, 95% CI [−0.368, 0.268]), conditioning (*t*(37) = -.731, *p* = .469, Cohen's* d* = −0.119, 95% CI [−0.437, 0.201]) or extinction (*t*(37) = -.462, *p* = .647, Cohen's* d* = −0.075, 95% CI [−0.393, 0.244]). Repeated-measures ANOVA showed no effects of stage (*F*(1.38, 50.94) = 2.276, *p* = .129, *η*_*p*_^2^ = .058), CS type (*F* <1), or interaction (*F* <1).

### Unconditioned stimulus (US) expectancy ratings

A 2 (stage: conditioning, extinction) × 2 (CS: CS^+^, CS^-^) repeated-measures ANOVA was conducted to analyze the expectancy ratings. Results showed no significant main effect of stage (*F*(1, 37) = .076, *p* = .784, *η*_*p*_^2^ = .002, 95% CI [−0.208, 0.274]). There was, however, a significant main effect of CS type (*F*(1, 37) = 5.023, *p* = .031, *η*_*p*_^2^ = .120, 95% CI [0.073, 1.445]), suggesting that CS^+^ images elicited higher expectations overall. Importantly, the interaction between stage and CS type was not significant (*F* < 1), indicating that the difference between CS^+^ and CS^-^ expectations did not vary across stages.

### Eye movements

At the US exposure stage, there was no significant difference in oculomotor avoidance between US^+^ and US^-^ (*t*(38) = −2.191, *p* = .035, Cohen’s* d* = −0.351, 95% CI [−0.672, −0.025]); however, a significant difference emerged during the conditioning phase (*t*(38) = −2.339, *p* = .025, Cohen’s* d* = −0.375, 95% CI [−0.697, −0.047]). That is, during the conditioning phase, participants demonstrated a significant oculomotor avoidance of US^+^ compared to US^-^ (Bonferroni correction applied, *p* = 0.025). This suggests that oculomotor avoidance emerged as a UR during the conditioning phase but was not present at the US exposure stage.

Analysis for pre-conditioning, conditioning and extinction stages showed that participants looked at the CS^+^ and CS^-^ images for the same amount of time at pre-conditioning (*t*(38) = -.707, *p* = .484, Cohen’s* d =* −0.113, 95% CI [−0.427, 0.202]), and at conditioning (*t*(38) = −1.865, *p* = .070, Cohen’s *d =* −0.299 95% CI [−0.618, 0.024]). However, they looked at CS^+^ less than CS^-^ at extinction stage (*t*(38) = −2.695, *p* = .010, Cohen’s *d =* .432, 95% CI [−0.757, −0.101]). That is, oculomotor avoidance emerged as a CR only at the extinction stage. While CS^+^ and CS^-^ did not differ at the pre-conditioning and the conditioning stage, the difference emerged at the extinction stage.

Additional analyses were conducted to test different questions. One of these questions was how oculomotor avoidance changed from conditioning to extinction, which was tested with a 2 (stage: conditioning and extinction) × 2 (CS type: CS^+^ and CS^-^) repeated-measures ANOVA. Results showed that both stage (*F*(1, 38) = 20.854, *p* < .001, *η*_*p*_^2^ = .354, 95% CI [−255.665, −98.613]) and CS type (*F*(1, 38) = 7.695, *p* = .009, *η*_*p*_^2^ = .168, 95% CI [112.012, 717.079]) had significant main effects. That is, the total amount of dwell time was larger during the conditioning than at the extinction stage,^5^ and participants looked at CS^+^ less than CS^-^. On the other hand, the interaction was not significant (*F(*1, 38) = .044, *p* = .835, *η*_*p*_^2^ = .001). However, general linear model analyses with Bonferroni-corrected pairwise comparisons revealed no significant difference in fixation duration between CS^+^ and CS^-^ stimuli during the conditioning phase (*F*(1, 38) = 3.480, *p* = .070, *η*_*p*_^2^ = .084), but significantly less time was spent looking at CS^+^ stimuli during the extinction phase (*F*(1, 38) = 7.262, *p* = .010, *η*_*p*_^2^ = .160). This finding suggests a potential trend, but it should be interpreted cautiously, as the non-significant interaction indicates that these differences may not be consistent across all levels of the factors. Future studies with larger sample sizes or additional conditions may help clarify these effects.

Another question was whether evaluative conditioning occurred with category conditioning. The 14-s presentation time in the conditioning stage was divided into 1-s segments to test whether CS acted as a signal for the US or as a stimulus that gains emotional value due to the conditioning process (see Armstrong et al., 2019). If the participants started to look less at the CS^+^ compared to CS^-^ towards the end of the 6-s CS presentation duration, we could conclude that CS^+^ acted as a signal. On the other hand, if participants looked at CS^+^ stimuli less at the earlier seconds of that duration, we could infer that these stimuli gained disgust-related aversive value (hedonic value) and caused a CR on their own (i.e., evaluative conditioning).

In order to see how CR changed over time, the first 6 s of the morph video presentation were tested in 1-s segments with a 2 (CS type: CS^+^ and CS^-^) × 6 (Segment: 0–1, 1–2, 2–3, 3–4, 4–5, 5–6) repeated-measures ANOVA. Results showed that CS type did not significantly affect dwell time (*F*(1, 38) = 3.314, *p* = .077, *η*_*p*_^2^ = .080, 95% CI [−7.091, 133.582]). The segment main effect was significant (*F*(2.216, 84.208) = 250.007, *p* < .001, *η*_*p*_^2^ = .868): dwell time was shorter in the first segment than in other segments.

There was a significant interaction between CS type and segment (*F*(3.61, 137.04) = 4.174, *p* = .004, *η*_*p*_^2^ = .099). General linear model analyses with Bonferroni-corrected pairwise comparisons showed that there was no significant difference between CS^+^ and CS^-^ in 0–1 (*F*(1, 38) = .146, *p* = .704, *η*_*p*_^2^ = .004) and 1–2 (*F*(1, 38) = 3.062, *p* = .088, *η*_*p*_^2^ = .075) segments, but the difference was observed in 2–3 (*F*(1, 38) = 4.266, *p* = .046, *η*_*p*_^2^ = .101), 3–4 (*F*(1, 38) = 5.733, *p* = .022, *η*_*p*_^2^ = .131), and 4–5 (*F*(1, 38) = 3.846, *p* = .057, *η*_*p*_^2^ = .092, marginal significance) segments. More interestingly, there was no difference between CS types at the 5–6 segment (*F*(1, 38) = 1.566, *p* = .218, *η*_*p*_^2^ = .040). That is, CS^+^ images were not just a signal for US^+^; instead, as evaluative conditioning suggests, they acquired disgust value and induced oculomotor avoidance on their own. As the transformation time approached, participants started to look at the CS^+^ image and tried to see the upcoming disgust scene.

**Fig. 3:**
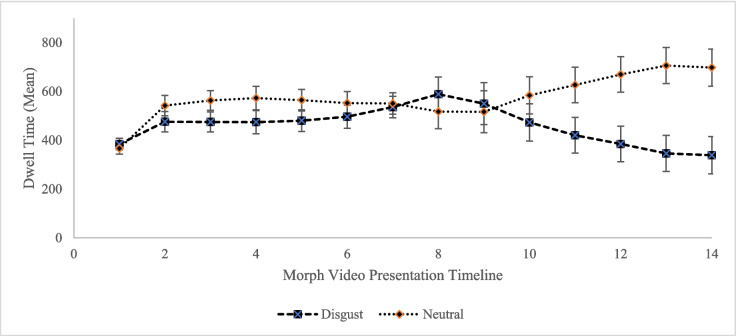
Evaluative conditioning analysis. Error bars represent standard error

The last 6 s of the morph video presentation were tested in 1-s segments to see how UR changed over time (Fig. 3). Results showed that US type’s main effect was significant (*F*(1, 38) = 6.896, *p* = .012, *η*_*p*_^2^ = .154, 95% CI [35.889, 277.437]), with US^+^ being looked at less than US^-^. Also, the time segment had a marginally significant main effect (*F*(2.741, 104.171) = 2.715, *p* = .053, *η*_*p*_^2^ = .073). However, follow-up tests showed no significant differences (all *p*’s > .05). There was a significant interaction between US type and segment (*F*(2.11, 80.215) = 19.119, *p* < .001, *η*_*p*_^2^ = .335). General linear model analyses with Bonferroni-corrected pairwise comparisons showed that US^+^ and US^-^ did not differ in terms of fixation duration in 8–9 (*F*(1, 38) = 0.546, *p* = .465, *η*_*p*_^2^ = .014) and 9–10 (*F*(1, 38) = .971, *p* = .331, *η*_*p*_^2^ = .025) segments, but US^+^ were looked at less than US^-^ in 10–11 (*F*(1, 38) = 6.018, *p* = .019, *η*_*p*_^2^ = .137), 11–12 (*F*(1, 38) = 13.238, *p* = .001, *η*_*p*_^2^ = .258), 12–13 (*F*(1, 38) = 19.566, *p* < .001, *η*_*p*_^2^ = .340), and 13–14 (*F*(1, 38) = 17.201, *p* < .001, *η*_*p*_^2^ = .312) segments. Although there was no oculomotor avoidance to US^+^ in the first 2 s, participants displayed increased avoidance throughout the presentation. The initial 2 s may be utilized to gather information about the disgusting scenes (Fig. 3).

## Discussion

The present study employed eye-tracking methodology to assess the effectiveness of the category-conditioning procedure in eliciting oculomotor avoidance, both as an unconditioned response (UR) and as a conditioned response (CR), within the context of disgust learning. Participants exhibited oculomotor avoidance to disgusting images (US^+^) as an UR and to neutral objects paired with those images (CS^+^) as a CR. Results showed that category conditioning with disgust images effectively elicited a CR through the trial-unique presentation method. These findings highlight the effectiveness of category conditioning in disgust learning and raise important questions for further discussion.

Self-report ratings showed that, unlike valence, disgust and arousal ratings did not differ between CS^+^ and CS^-^ with the conditioning manipulation. From pre-conditioning to post-conditioning, participants evaluated all CSs more negatively, and regardless of the stage, they rated CS^+^ stimuli more negatively compared to CS^-^ stimuli. This discrepancy between self-report measures and eye-tracking results for disgust was unexpected and may have arisen due to inherent limitations of self-report measures. A previous study, which used a similar category-conditioning procedure without eye tracking, showed that CS^+^ stimuli elicited higher disgust values than CS^-^ stimuli after conditioning (Söylemez & Kapucu, 2024b). Since the present study is an eye-tracking experiment, and it required staying in a chin-rest position for a long time, the exhaustion the participants experienced might have affected their subjective evaluations. Moreover, previous disgust conditioning studies that used eye-tracking methods indicate that self-report measures can cause different results, and they are not a reliable indicator for disgust conditioning as much as eye tracking (for review, see Armstrong et al., 2021).

Although valence and disgust ratings suggest that neutral stimuli acquired aversive but not disgust value, this does not necessarily indicate that they failed to gain disgust-specific characteristics. The oculomotor avoidance pattern observed in the study aligns with prior research indicating that disgust – but not fear – is uniquely associated with avoidance tendencies (Armstrong et al., 2014). Furthermore, it has been shown that the disgust stimuli used elicit significantly higher disgust than fear (see Söylemez & Kapucu, 2024b). The study by Armstrong and colleagues (2014), which utilized a disgust-conditioning procedure and is referenced in this paper, showed that CS^+^ stimuli received higher disgust ratings following conditioning. At this point, it is important to highlight a key difference between the aforementioned study and the current one: while the current study employed category conditioning with trial-unique presentations, Armstrong and colleagues (2019) used repeated presentations of the same stimuli. This methodological difference may have allowed participants in their study to provide evaluations more easily. Therefore, rather than being a mere reflection of general aversive learning, the findings of the present study may be indicative of disgust-specific associative processes. However, to definitively rule out this claim, future studies should test another aversive emotion, such as fear, using the same experimental procedure with eye-tracking methodology. Lastly, there were no differences between CS types for arousal after conditioning. Carretié and colleagues (2011) suggest that there is no risk of attack in the environment in the case of disgust, and this situation may create no arousal differentiation for disgust-associated stimuli. Finally, according to the expectancy ratings, participants learned the CS-US contingency independent of the stage. Stage-independent contingency learning is consistent with previous studies (Armstrong et al., 2014; Mason & Richardson, 2010; Wang et al., 2021), which argued that disgust learning is resistant to extinction.

Results supported the idea that disgust learning relies on evaluative conditioning: participants avoided looking at the CS^+^ images during the middle seconds of the presentation; however, this avoidance disappeared in the final second. Thus, instead of being a signal for the US^+^, CS^+^ acquired the disgust value and led to oculomotor avoidance (Armstrong et al., 2019). This finding highlights the distinctive nature of the CS-US relationship in disgust learning. Unlike other forms of Pavlovian conditioning, evaluative conditioning fundamentally transforms the CS by altering its intrinsic affective value. Rather than merely signaling the presence of the US, the CS in evaluative conditioning becomes an emotionally charged stimulus itself, integrating into a unified affective representation with the US (Köksal et al., 2017). Crucially, this does not mean that in signal learning the CS fails to acquire the emotional valence of the US; rather, the key distinction lies in the depth of this transformation. In evaluative conditioning, the CS does not just predict the US but undergoes a qualitative shift, acquiring an independent emotional significance that persists even in the absence of the US. This distinction has been widely supported in the literature (De Houwer et al., 2001; Hofmann et al., 2010; Lipp et al., 2003), reinforcing the idea that evaluative conditioning leads to more robust and persistent affective learning than traditional signal learning. Consequently, US devaluation, which typically affects CRs in other forms of Pavlovian conditioning, does not influence the CRs in disgust learning (Mertens et al., 2021).

Taken together, the results suggest that CS-US contingency was reported regardless of the stage of expectancy measures and that CR emerged both at conditioning (evaluative conditioning analysis) and more strongly at the extinction stage. In other words, CR resisted the extinction phase. One of the reasons for this resistance might be related to the fact that disgust learning is based on evaluative conditioning (Armstrong et al., 2014; Dalmaijer et al., 2021; Olatunji et al., 2007). Another reason might be attentional avoidance, as it prevents extinction and preserves disgust learning by limiting exposure to corrective information and hindering cognitive reappraisal of established associations (Cisler & Koster, 2010; Mogg et al., 1987). While vigilance-related responses, such as sustained attention to a stimulus, have been shown to resist extinction (Armstrong et al., 2022a, b), our findings suggest that oculomotor avoidance, rather than vigilance, characterizes responses to CS^+^ stimuli. This avoidance may contribute to the persistence of disgust learning by reinforcing attentional biases and preventing habituation. Moreover, the cognitive impenetrability of disgust can lead a person to have disgust reactions and avoidance responses to certain stimuli despite knowing that they are irrational and illogical (Royzman & Sabini, 2001). However, this study supported the view that curiosity competes with attentional avoidance through the conditioning process (Armstrong et al., 2014, 2019), and demonstrated that curiosity is a good motivation for intervention in disgust learning (Crabtree & Hampson, 2019). As disgust is an emotion with information-based content (Turner & Silvia, 2006), it arouses interest (Silvia, 2006) and curiosity (Oosterwijk, 2017).

While curiosity was not directly measured in this study, prior research suggests that disgust can evoke curiosity under certain conditions (Tybur et al., 2013). Future studies could investigate this relationship more systematically by incorporating direct curiosity assessments. Enhancing curiosity as a form of intrinsic motivation may provide a way to overcome the inherent rigidity of disgust. The inability of disgust to habituate and its persistent tendency to elicit avoidance, even in the presence of external rewards (Dalmaijer et al., 2021), suggest – based on the findings of this and previous studies – that curiosity could serve as an effective internal counteracting force. Using eye-tracking methodology in this study to assess the success of disgust conditioning not only enables the investigation of disgust learning but also facilitates the exploration of other cognitive processes related to disgust. The eye-tracking method provides opportunities to interpret attentional processes because eye movements are shown as a direct measure of overt attention (Olatunji et al., 2017). Previous eye-tracking studies demonstrated that there is no automatic attentional capture by disgust stimuli, and attentional avoidance to these stimuli emerges in a controlled manner in subsequent processing stages (Armstrong et al., 2014, Armstrong et al., 2012; Bradley et al., 2015; Cisler et al., 2009b; Mason & Richardson, 2010). Similarly, disgust was reported to generate less attentional bias (automatic attentional capture) compared to fear when conditioned stimuli were involved (Wang et al., 2021). In the present study, we found no differentiation in the eye-movement data regarding dwell times between CS^+^ and CS^-^ in the earlier seconds, but CS^+^ was looked at less than CS^-^ in the following seconds. In other words, CS^+^ items did not attract more attention but instead caused attentional avoidance later in processing.

Although this study has succeeded in testing its proposed hypotheses and achieving its intended objectives, it should be evaluated considering some limitations. First, the results showed the sample size was not sufficient. Although our power analysis indicated that the sample size was sufficient, the observed effect sizes were smaller than anticipated. Additionally, the novel paradigm used in this study and the lack of repeated exposures (unlike the study by Armstrong and colleagues (2019)) may have limited the accumulation of statistical power, potentially contributing to the observed results. Future studies with larger samples may help capture more subtle effects under these conditions. Second, this study was conducted with only university students, yet disgust is argued to be an age-sensitive emotion (Curtis et al, 2004). Third, in this study, only the disgust emotion was used, and neutral scene images were used for comparison purposes. To support the specificity of the findings to disgust, another discrete emotion (e.g., fear) could be used for comparison. Addressing these limitations in future studies will enrich the literature on this topic. Despite these limitations, this study is believed to make a significant contribution to the literature on disgust learning. For example, this procedure can be utilized to investigate the processes of episodic memory related to disgust within the context of conditioning. A recent study demonstrated that category conditioning enhances episodic memory for disgust-associated stimuli but does not have the same effect for fear-associated stimuli (Söylemez & Kapucu, 2024b). In summary, embracing this broader perspective can yield deeper insights into the mechanisms underlying disgust learning.

In conclusion, the current study confirmed that the category-conditioning procedure with the selected stimuli successfully assigned a disgust value to neutral stimuli, much like a procedure involving repeated presentations by Armstrong and colleagues (2019) and a procedure involving shock (Dunsmoor et al, 2014). It is believed that the findings from this study will benefit future research on disgust and contribute to considering the learning-based structure of it. Although disgust conditioning has received less attention than fear conditioning until recently, its robust and extinction-resistant nature can contribute to maladaptive outcomes on both individual and societal levels (Söylemez & Kapucu, 2024a). Therefore, gaining a deeper understanding of this emotion is a crucial objective in psychological research. Trial-specific presentations enable a more comprehensive investigation of disgust learning, facilitating the simultaneous examination of multiple cognitive processes such as learning, attention, and memory. This paradigm could be further refined by incorporating memory assessments at different time intervals, integrating various emotional conditions, or exploring how attentional engagement during the learning phase under different emotional contexts shapes subsequent memory performance. Thus, it is hoped that this study contributes to future research on disgust learning by simultaneously benefiting from the richness of different literatures within the same design.

## Data Availability

All data, supplementary materials, and ethics approval are publicly available via the Open Science Framework at: https://osf.io/rp8gc/

## Footnotes

1 Disgust ratings were assessed on a 7-point scale, ranging from 1 (very low) to 7 (very high).

2 Example disgust stimuli can be found at the following link: https://osf.io/rp8gc/.

3 Typicality ratings were assessed on a scale between 1 (not at all) and 10 (very much). Detailed information about the stimuli is available on https://osf.io/rp8gc/

4 Expectation measurement was not given in the pre-conditioning stage to prevent participants from having prior knowledge about the content of the experiment or making premature predictions before the manipulation.

5 This effect might emerge because morph video stimuli were used in the conditioning stage, while static image stimuli were used in the extinction stage.

## Code availability

No code was used in this study that would require sharing.
